# Supplementary figures and images for: IgaA negatively regulates the Rcs Phosphorelay via contact with the RcsD Phosphotransfer Protein
Source: PLoS Genet. 2020 Jul 27;16(7):e1008610. doi: 10.1371/journal.pgen.1008610 (PMC7418988; doi:10.1371/journal.pgen.1008610)

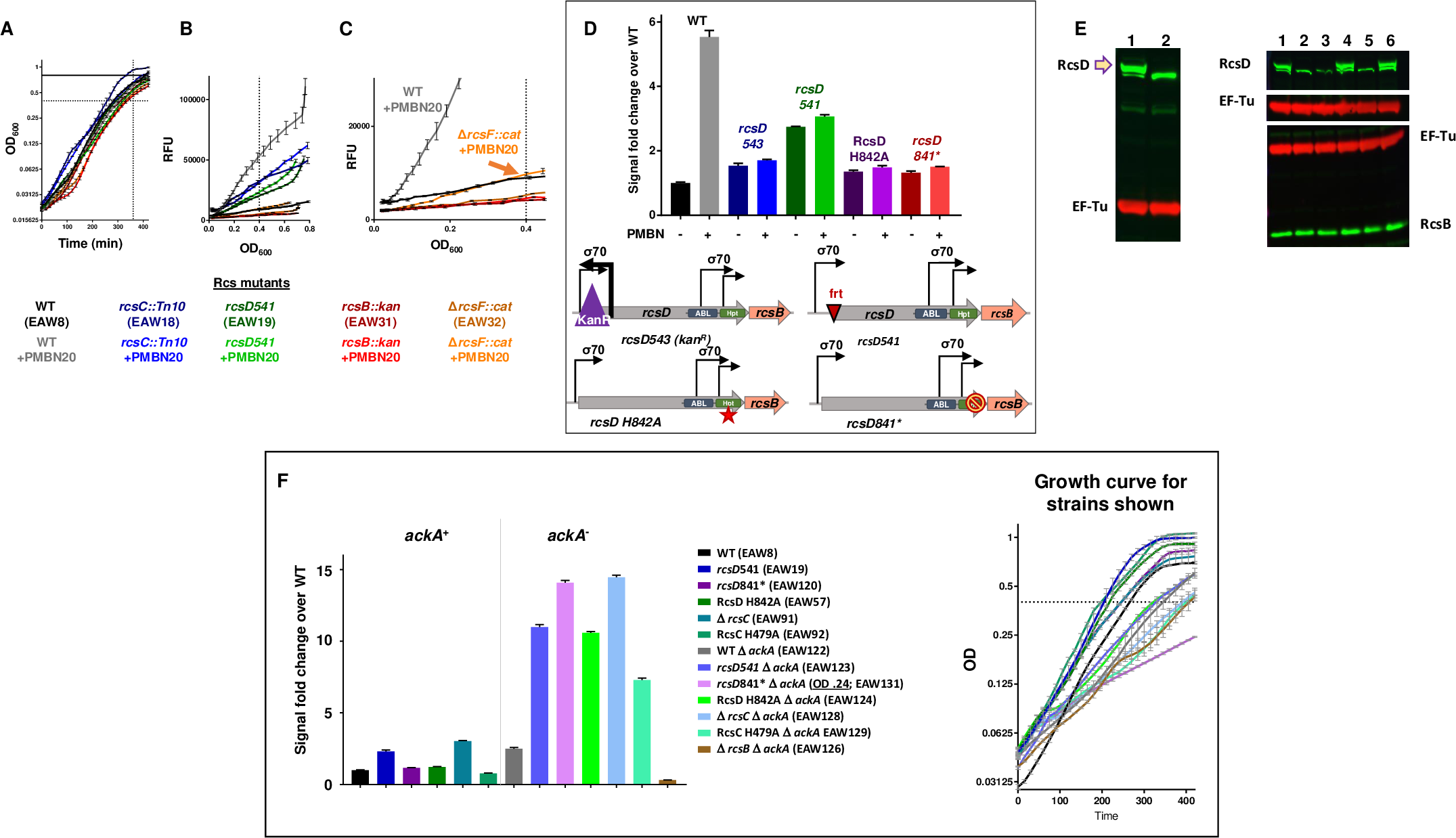

Supplement: S1 Fig — S1A-C use a similar color code as Fig 1B, but with two shades of each color to indicate growth with or without PMBN; strains and treatments are shown with their color code. A. Growth curve of each strain +/- PMBN 20 μg/mL as shown in Fig 1B. Dotted lines represent an OD600 of 0.4 (horizontal line) and a 360 min (6 hour) time point (vertical line), used as the standard measurements for fluorescent strains, unless otherwise indicated. Demonstrated in A is that stationary phase doesn’t begin for any strain until close to or after OD600 0.8 under these growth conditions. Stationary phase always induces Rcs and can cause buildup of cells in well bottoms; therefore, measurements were not made past OD600 0.8 (solid horizontal line in panel A). Throughout the figures, if a strain has a growth defect that does not allow it to reach OD600 0.4 before the 360 min time point, it is noted with its actual OD600 on the relevant bar graph legend. B. Relative fluorescent units (RFU) as a function of OD600 for strains used in Fig 1B. The vertical dotted line represents the measurement point that is shown in the Fig 1B bar graph, OD600 0.4. These traces demonstrate the overall differences in Rcs activation of each strain. The effect of PMBN on the slope of each line can be seen clearly. For example, WT without PMBN (black) has a low slope throughout the graph, while WT + PMBN (gray) has a noticeably higher slope. The rcsC or rcsD mutants (blue and green respectively), have slight differences in RFU between treated and untreated conditions at each growth point; these differences do not dramatically affect the overall slope of the trace, indicating that small fluorescence differences here do not represent activation of Rcs as a whole. When a strain stops growing (for instance, as with WT+PMBN, gray line at OD600 near 0.8) and the fluorescence continues to increase, the slope of the line becomes much sharper; we avoid using measurements in this range. C. Enlarged version of portion of S1B [file pgen.1008610.s001.tif]

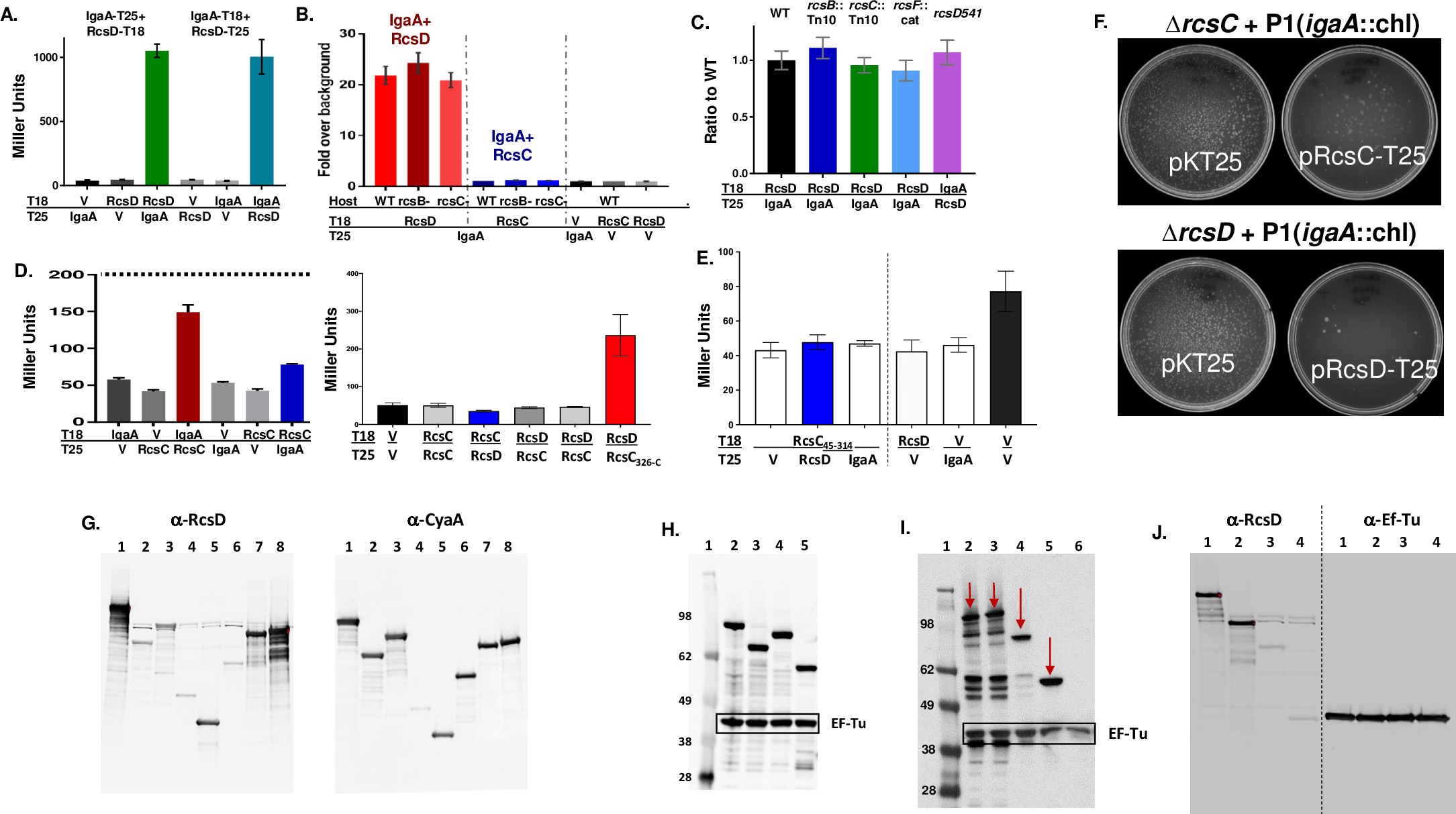

Supplement: S2 Fig — A. IgaA and RcsD interact well regardless of which tag is used on each. The interaction registers at least 1000 Miller units, while vector control experiments yield only 50, giving a 20-fold signal to noise ratio. Plasmids used: pEAW1 (IgaA-T18), pEAW2 (IgaA-T25), pEAW7 (RcsD-T25), pEAW8 (RcsD-T18). All error bars throughout the figures represent standard deviation. B. IgaA and RcsD interact robustly compared to control empty vectors, regardless of strain background. IgaA/RcsC interaction was below the limit of detection in all strains tested. Empty vector controls were performed in the WT background (BTH101), rcsB::Tn10 (EAW1), and rcsC::Tn10 (EAW2) and averaged to use as background. C. Interaction of RcsD and IgaA occur irrespective of strain background. Results from S2B merged with results from different experiments done in the rcsF- (EAW4) and rcsD- (EAW12) backgrounds. Each bar represents the relative IgaA/RcsD interaction measurement in the respective mutant host relative to the IgaA/RcsD interaction in wild type cells; this positive control is present for normalization in every assay of interaction of RcsD and IgaA wild type and mutants. D. RcsC interaction with IgaA or RcsD cannot be reliably detected irrespective of tag orientation. Left panel: IgaA/RcsC were fused in both orientations and tested in the BTH101 host. The dotted line at 200 Miller units represents approximately 4-fold over the background controls, the standard used in this work for a consistent, repeatable interaction determination. Note difference in beta-galactosidase values for even the strongest interaction here (150 Miller units) compared to the interaction of RcsD with IgaA (S2A Fig). Plasmids used: pEAW1 (IgaA-T18), pEAW6 (RcsC-T25), pEAW2 (IgaA-T25), and pEAW5 (RcsC-T18). V: vector, pUT18 for the T18 vector and pKNT25 for T25 vector. Right panel: RcsC-T18 (pEAW5) and RcsC-T25 (pEAW6) were tested for interactions with each other and with RcsD (RcsD-T25 (pEAW8) and RcsD-T18 (pEAW7)), bu [file pgen.1008610.s002.tif]

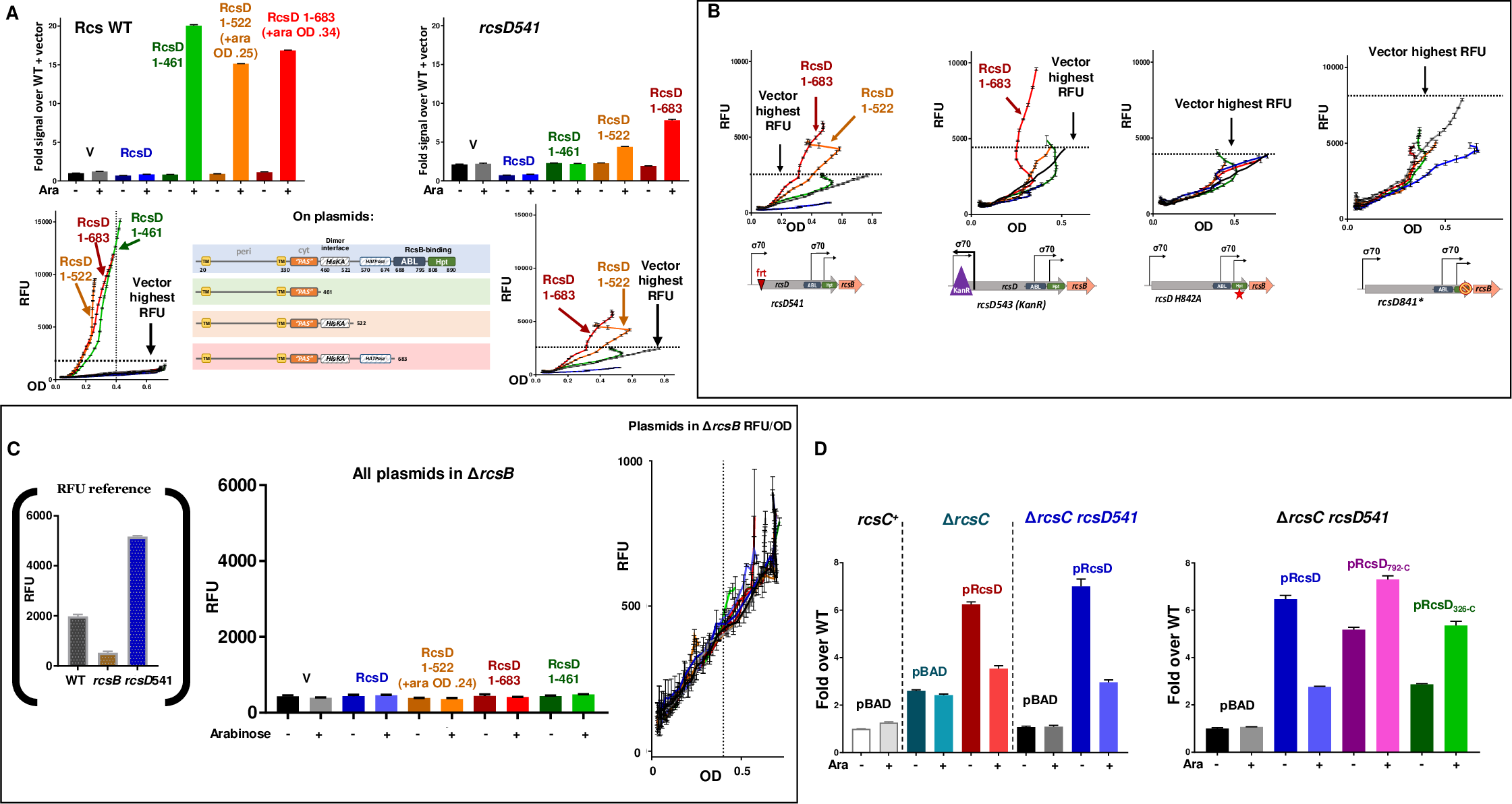

Supplement: S3 Fig — A. Signaling upon expression of RcsD N-terminal fragments. As for Fig 3A, with additional plasmids. RcsD C-terminal truncation constructs were expressed from arabinose-inducible plasmids in a WT (EAW8) and an rcsD541 (EAW19) host. The graphs of strain fluorescence (RFU) as a function of OD600 for cells grown with arabinose are presented below their respective bar graphs. Constructs are color-coded: black: V, (pBAD24); blue: RcsD+, (pEAW11); green: RcsD1-461 (pEAW11m2); orange: RcsD1-522 (pEAW11alpha); red: RcsD1-683 (pEAW11b). Note that a change in slope on the fluorescence/ OD600 graph demonstrates some level of PrprA-mCherry activation, and that the orange (RcsD1-522) and red (RcsD1-683) slopes are very different from other slopes in the rcsD541 strain. Cell lysis can be seen as a reduction in OD600 resulting in a leftward shift in the line (see orange and green lines in rcsD541 host). Note that, in spite of lysis for RcsD1-461 in rcsD541, greater fluorescence did not result, compared to the vector control in the same time period. Therefore, lysis does not automatically increase PrprA-mCherry fluorescence. Highest RFU with vector shown by horizontal dotted line, for comparison with experimental curves. This data and results in S3B are further discussed in S1 Text. B. Activity of RcsD plasmids in different rcsD mutants. Based on the unexpected signal from plasmids lacking the Hpt domain in rcsD541 (S3A Fig), three additional rcsD alleles were tested with RcsD C-terminal truncation plasmids. Fluorescence as a function of OD600 is shown for cells grown with arabinose, as in S3A Fig, but in strains carrying the four different chromosomal rcsD alleles, rcsD541 (EAW19, repeated from S3A Fig), rcsD543 (EAW9), rcsDH842A (EAW57) and rcsD841* (two stop codons at residue 841, EAW120), as previously studied without plasmids in S1D Fig. Each rcsD allele is shown as an inset below the Fluorescence/ OD600 trace for that strain. Plasmids are color-coded as in S3A Fig. Highest RFU [file pgen.1008610.s003.tif]

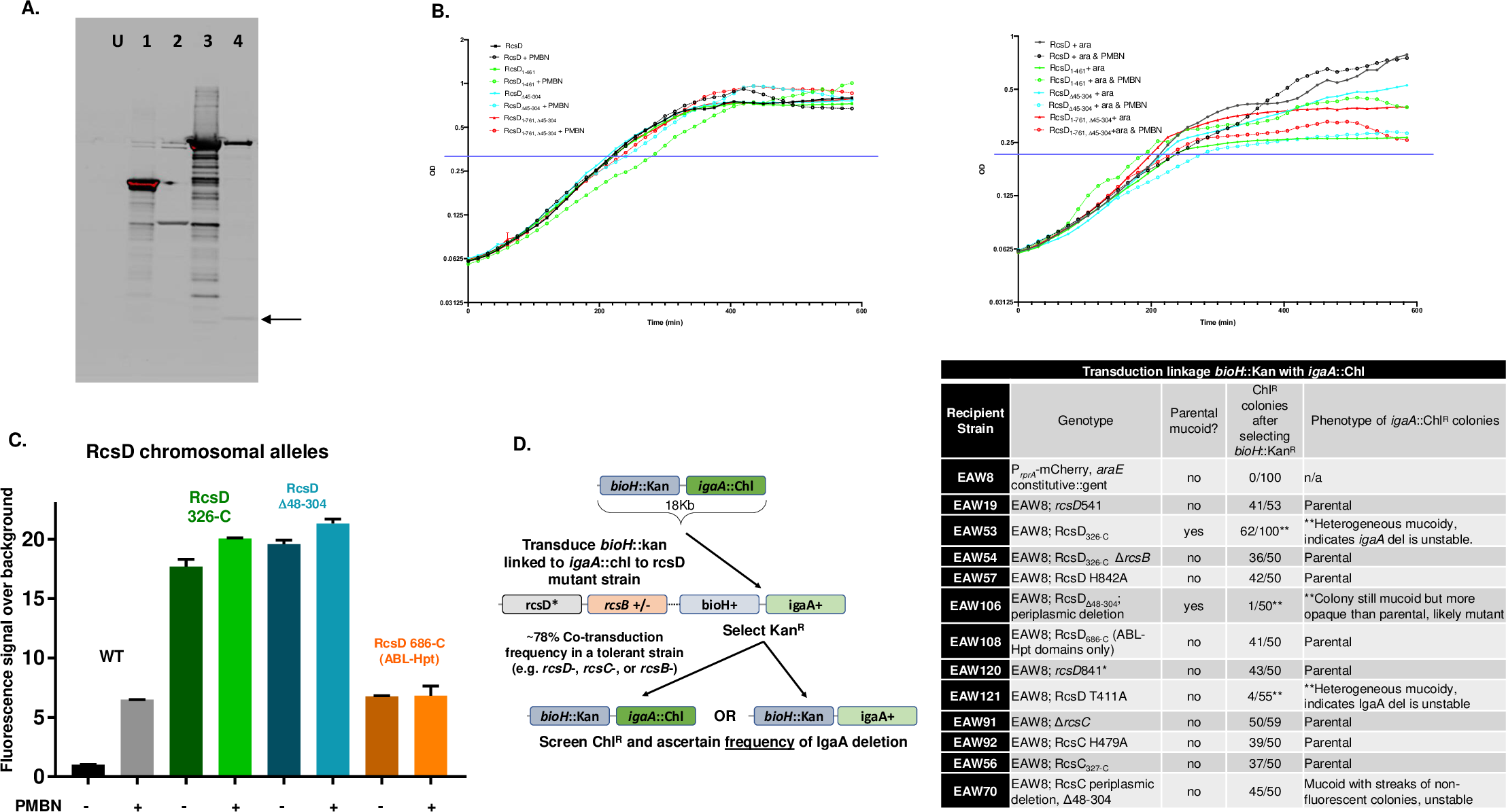

Supplement: S4 Fig — A. Western blot for expression of pRcsD truncated proteins from the pBAD promoter tested in Fig 3C. Cells carrying pBAD-RcsD plasmids were grown in LB ampicillin (100μg/ml) with 1% glucose to an OD600 of 0.3, washed and resuspended in LB ampicillin with 0.02% arabinose and allowed to grow for 2 hr before sample collection. Samples were run on a 4–12% gradient gel in MOPS buffer for 1 hr and probed with anti-RcsD antibody. Lane 1: RcsDΔ45–304, 632 aa (pEAW11 peri); Lane 2: RcsD1-461, 458 aa (pEAW11m2); Lane 3: RcsD, 890 aa (pEAW11); Lane 4: RcsD1-461, Δ45–304, 174 aa (indicated with arrow). Note that the antibody preferentially detects RcsD derivatives carrying the Hpt domain, not present on the proteins in lanes 2 and 4 (see S2G Fig) B. Growth of the strains used in Fig 3C. Cell growth in glucose in the presence or absence of PMBN (left panel) or in the presence of arabinose, but in the presence or absence of PMBN (right panel) are shown. RFU units for cells that had grown to OD600 of O.3, shown with a horizontal line on the graphs, were used to create the graph in Fig 3C. C. Signal activation and PMBN response for chromosomal mutants of RcsD. rcsD alleles were introduced into the chromosomal rcsD locus to create: rcsD326-C (EAW53), rcsDΔ48–304 (EAW106) and rcsD686-C (EAW108), grown and assayed as in Fig 1B. rcsD792-C could not be introduced without deleting promoters for RcsB, so that construct was not made. These alleles performed as their plasmid counterparts did, with the longer constructs roughly equivalent in their high signal and slow growth and the rcsD686-C allele appearing less efficient at passing signal to RcsB. Only the rcsD686-C allele can tolerate an igaA deletion (S4D Fig). D. Co-transduction of igaA::chlR with bioH::kan as an assay of Rcs function. Schematic shows igaA::chlR cotransduction frequency experiment using linked bioH::kan. The bioH::kan igaA::chlR P1 donor (EAW66) was constructed in an rcsD541 mutant. The table lists frequency of igaA::ch [file pgen.1008610.s004.tif]

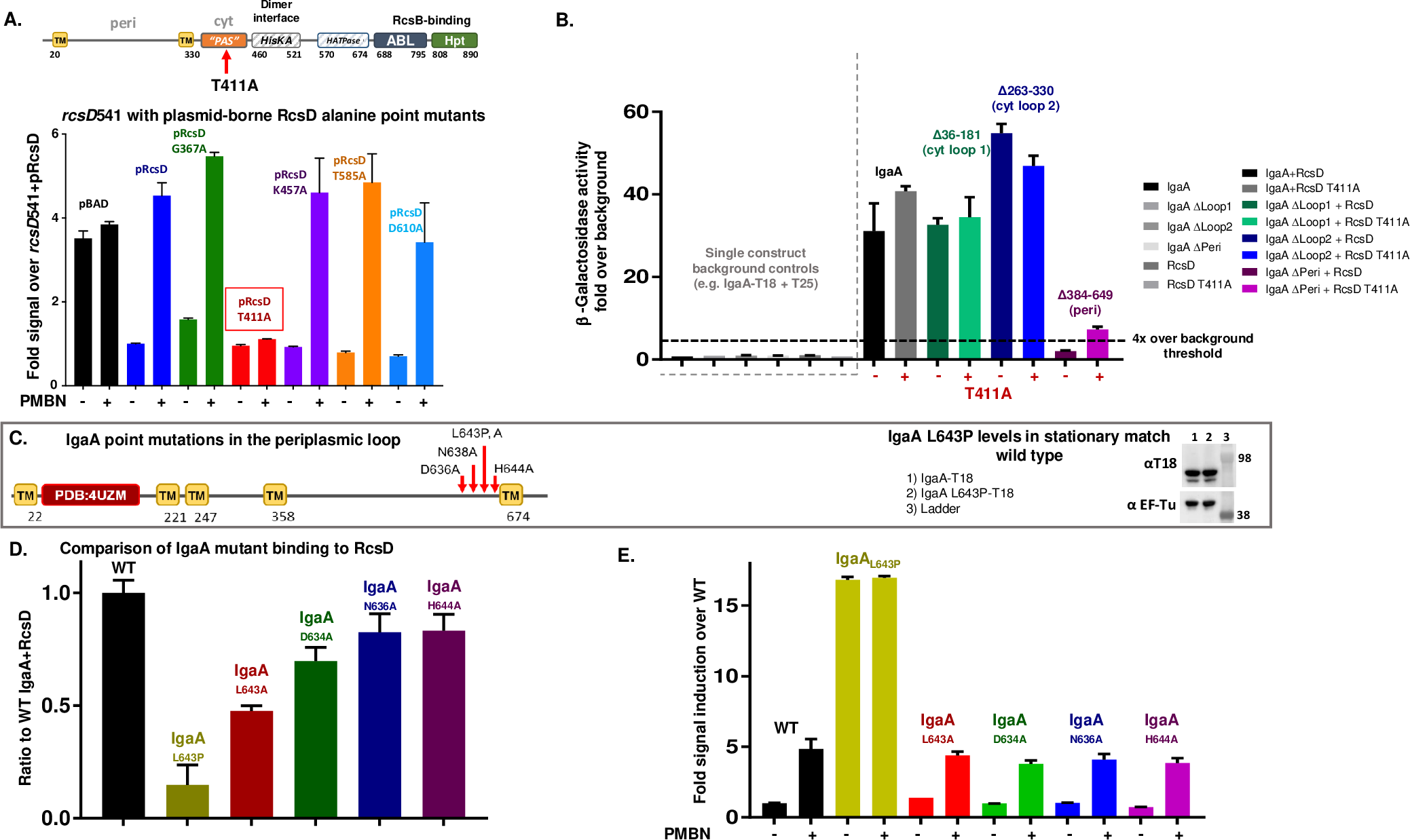

Supplement: S5 Fig — A. Plasmid-borne alleles in the RcsD cytoplasmic domain that retain phosphatase function. Strain EAW19 (rcsD541) with mutant derivatives of pBAD24-RcsD (pEAW11) were grown in MOPS glucose or MOPS glucose with PMBN, all with ampicillin and without arabinose induction and assayed for PrprA-mCherry expression during growth. rcsD541 has a higher signal than wild type and can be complemented with WT RcsD on a plasmid (compare lanes 1 and 3). The RcsD+ construct responds to PMBN, unlike empty vector (compare lanes 1 to 2 and 3 to 4). Plasmids encoding rcsD alanine mutations in the cytoplasmic domains were screened for those that complemented rcsD541, reducing the basal level of expression; these were then assayed with and without PMBN. Of the 5 alleles shown here, 4 were inducible with PMBN. However, although it complements rcsD541, significantly lowering PrprA-mCherry signal, interpreted as evidence of phosphatase activity, expression of the rcsDT411A point mutant was not induced in response to PMBN. B. BACTH IgaA loop deletion interactions with RcsD WT vs RcsD T411A. Experiment is as in Fig 4B, but showing additional controls. The IgaA+RcsD constructs gave β-galactosidase levels greater than thirty-fold over the single construct (background) controls. T18 derivatives carrying IgaA cytoplasmic loop one deletion (Δ36–181, cyt loop 1; pEAW1cyt1), IgaA cytoplasmic loop two deletion (Δ 263–330, cyt loop 2; pEAW1cyt2), IgaA periplasmic loop deletion (Δ 384–649, peri; pEAW1peri) were tested with RcsD-T25 WT (pEAW8) or RcsD T411A (pEAW8T); all were assayed in BTH101. C. IgaA point mutations in the periplasmic loop. Schematic showing point mutations surrounding IgaA L643P, a mutant of IgaA defective in Rcs negative regulation. In a western blot using the anti-T18 Cya antibody, the level of the T18-IgaA fusion protein was similar for L643P and wild type IgaA, ruling out protein instability as the explanation for its loss of function. EF-Tu was used as a loading control. Plasmids [file pgen.1008610.s005.tif]

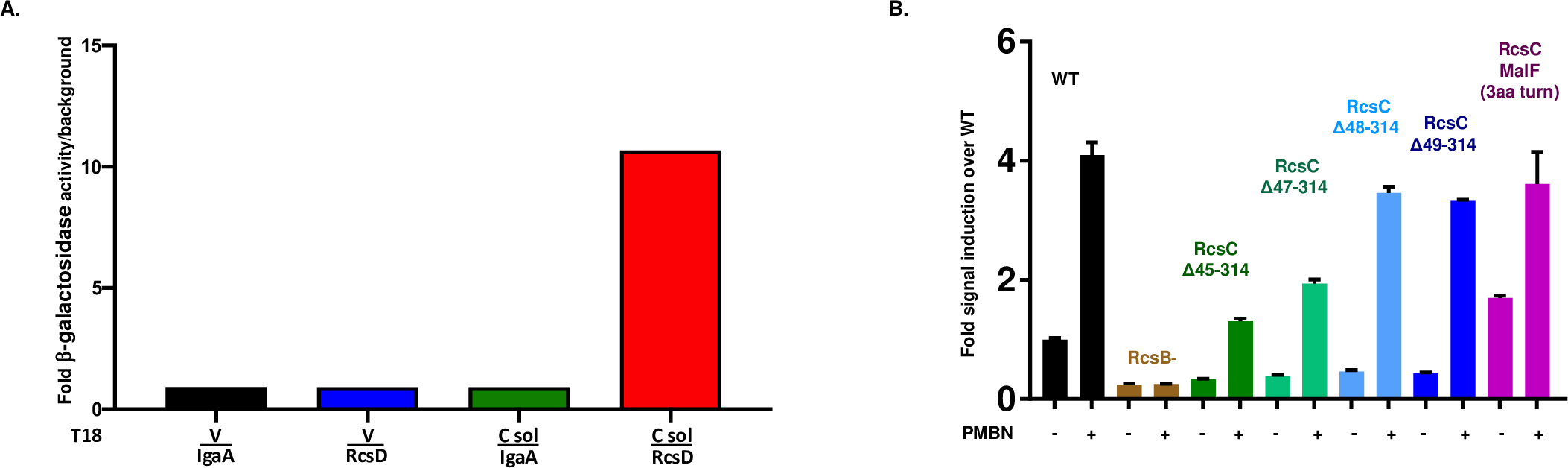

Supplement: S6 Fig — A: Bacterial two-hybrid assay of interaction of cytoplasmic portion of RcsC with RcsD but not IgaA. Plasmids used: IgaA-T25 (pEAW2), RcsD-T25 (pEAW8), and RcsC sol (RcsCC326-C-T18, pEAW5s). B: Assays of RcsC periplasmic deletions and chimeric RcsC. RcsC periplasmic deletions perform differently when exposed to PMBN, depending on the linker length between transmembrane domains and the identity of those transmembrane domains. Strains present include (L to R) EAW8 (WT), EAW31 (rcsB::kan)), EAW61(rcsCΔ45–314), EAW69 (rcsCΔ47–314), EAW70 (rcsCΔ48–314), EAW71 (rcsCΔ49–314), and EAW72 (rcsCMalF). (TIF) [file pgen.1008610.s006.tif]

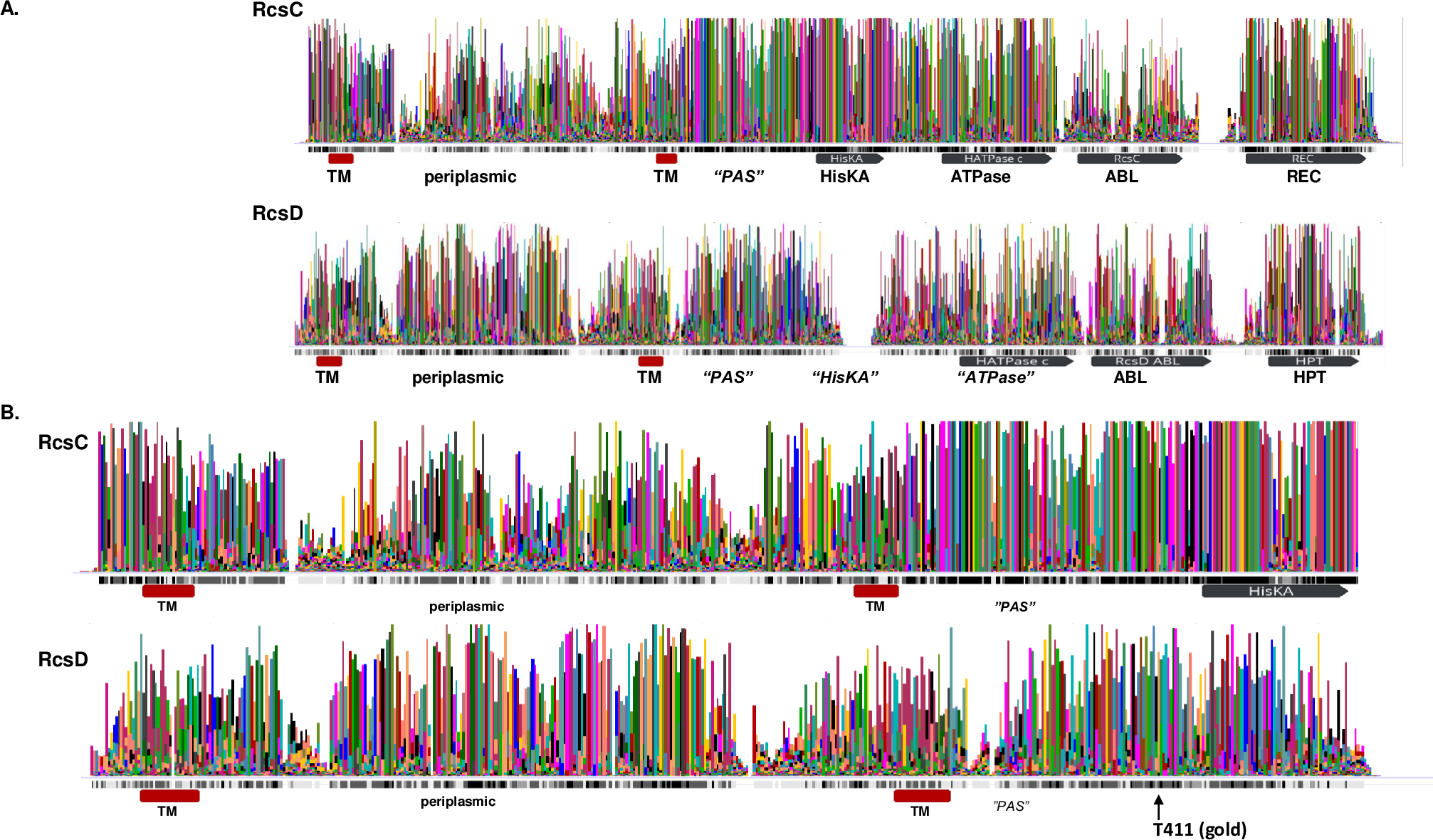

Supplement: S7 Fig — A. Sequence alignments of 251 RcsC and RcsD homologs in Enterobacterales demonstrate differing regions of amino acid conservation. Protein sequences were manually collected from 251 Enterobacterales species using NCBI Taxonomy Browser to determine genera for inclusion. Each genus in Enterobacterales was checked for species containing annotated adjacent RcsC and RcsD ORFs on their genomes using NCBI Protein and Nucleotide; species containing full sequences of the region were selected for inclusion in the alignment. RcsC and RcsD alignments were performed using ClustalW and the sequence logos were automatically generated in Geneious. High amino acid conservation is demonstrated by high lines in the logo and dark regions in the line below the logo, which represents a consensus sequence. Transmembrane regions are marked by red rectangles, domain regions are marked by black arrows. B. Expanded alignment for the Trans-membrane and initial cytoplasmic regions of RcsC and RcsD. The location of T411A in the PAS-like domain of RcsD is shown with an arrow. (TIF) [file pgen.1008610.s007.tif]
