# Supplementary material for: IgaA negatively regulates the Rcs Phosphorelay via contact with the RcsD Phosphotransfer Protein
Source: PLoS Genet. 2020 Jul 27;16(7):e1008610. doi: 10.1371/journal.pgen.1008610 (PMC7418988; doi:10.1371/journal.pgen.1008610)
Supplement: S1 Text — (DOCX) [file pgen.1008610.s009.docx]

Supporting Text:

**S1 Text: A possible role for the RcsD Hpt domain in the unexpected activation of the phosphorelay with certain RcsD mutant proteins**

Our understanding of signaling in the Rcs phosphorelay is that the only active histidine kinase in this system is RcsC; phosphate moves from RcsC to RcsD and then to RcsB, as seen in Fig 1A, resulting in activation of the P_rprA_-mCherry reporter. Other sources of phosphorylation might include small molecules, such as acetyl phosphate, capable of directly phosphorylating RcsB (as in S1F Fig), or potentially cross-talk from other histidine kinases, possibly via the RcsC response regulator domain or directly to RcsB. In the absence of RcsC or RcsD, these alternative sources of phosphorylation provide low level activation of the phosphorelay (Fig. 1B).

In S3A Fig, induction of plasmids expressing truncations RcsD_1-522_ and RcsD_1-683_, both missing the Hpt domain, were able to significantly increase signal in the *rcsD*541 host. However, these same plasmids caused lysis in WT and *rcsD*541 cells (S3A Fig). These constructs contain regions of RcsD that are not well understood, including an ancestral histidine kinase structure between residues 462 and 683. To further investigate the basis for this unexpected signaling, the same plasmids were tested in three additional *rcsD* mutants (S3B Fig). In a strain with a chromosomal mutation in the phosphotransfer active site, RcsD H842A,or in a strain containing a stop codon at residue 842, right before the active site (*rcsD*841*), the RcsD_1-522_ and RcsD_1-683_ truncations did not raise the level of P_rprA_-mCherry, suggesting that the nature of the chromosomal *rcsD* mutation is contributing to the effect caused by overproduction of the fragments (S2B Fig). An intact Hpt domain in the chromosomal copy of *rcsD* appears to be necessary to allow this modest activation, possibly suggesting that the *rcsD*541 mutant may express a low level of the Hpt domain. This signaling is fully dependent upon RcsB (S3C Fig).

A second suggestion for expression of an Hpt domain, separate from some regions of RcsD, was the observation, not yet pursued, of high phosphorelay expression from plasmids of RcsD with unexpected stop codons upstream of the ABL and Hpt domains. These plasmids, rather than acting like nulls, had activity significantly above that of a null strain, again suggesting that they might be expressing a C-terminal fragment of RcsD. Whether this is ever made under wild-type physiologically relevant conditions remains to be determined but would provide the possibility of an IgaA-resistant signaling pathway. Thus far, we have not detected evidence of such an expressed domain of RcsD, and further work on this remains for the future.
