# Supplementary material for: IgaA negatively regulates the Rcs Phosphorelay via contact with the RcsD Phosphotransfer Protein
Source: PLoS Genet. 2020 Jul 27;16(7):e1008610. doi: 10.1371/journal.pgen.1008610 (PMC7418988; doi:10.1371/journal.pgen.1008610)
Supplement: Attachment — Submitted filename: Response to the Reviews and Major Changes 5_17_20 final.docx [file pgen.1008610.s013.docx]

Response to the Reviews and Major Changes, Wall et al

Many thanks for handling our article and for your patience as we have revised this in response to the reviewers’ comments. We have added additional data in response to reviewers’ comments, taken out a few parts that seemed less important and distracting, and done substantial rewriting as a result of these changes.

Major changes and other issues; detailed responses to the reviewers are below:

1) New data has been added to address some of the issues raised by reviewers:
   a. We have constructed a new RcsD derivative, deleted for the periplasmic region and containing only the PAS-like domain in the cytoplasmic region. In Figure 2, we used this to show that in the context of a membrane protein, the PAS-like domain is sufficient for interaction with IgaA (new panel 2C, accompanying Western blot, Fig. S2J). In Figure 3C, this construct was tested for ability to titrate IgaA; the results support both an important role for the periplasmic domain and reinforce our model for the PAS-like domain in signaling. These changes, done in response to reviewers’ queries, we believe further strengthen the paper.
   b. New panel in Supplemental figure S2G showing the quantitation of different RcsD truncations in the context of RcsD-T18 fusions, and ability to detect these with the anti-RcsD antibody, compared to detection with the anti-Cya antibody that detects the T18 tag.
2) Added a summary chart of phenotypes (new Fig. 3D).
3) Changed Fig. 6 to better summarize our model.
4) While we tried to experimentally address some of the other requests of the reviewers, in many cases we were unable to provide useful additional data. In the interests of not further complicating the paper, some of these explanations, including data, are provided to the reviewers below but not added to the manuscript. In particular, we find that there are significant limits in bacterial two hybrid experiments that preclude some suggested experiments of that type. Probably the most significant issue is that we were unable to provide the requested positive control for RcsC interactions, in spite of a variety of new experiments. The additional negative data has been added to the paper (Fig. S2D, E). That leads us to rephrase and soften our interpretations of negative results for RcsC interaction with IgaA.
5) Rewriting throughout to improve clarity, shorten text somewhat, and address all comments.
6) Sections have been moved out of the main text or significantly shortened in the main text; details are now either in supplemental figure legends, where appropriate, or in the newly added S1 Text. Some sections were removed as unnecessary and distracting, per reviewers’ comments that the paper was difficult to get through (deletion of panels S2B, S3E and S3F).

Reviewer's Responses to Questions

**Comments to the Authors:
Please note here if the review is uploaded as an attachment.**

Reviewer #1: The Regulator of Capsule Synthesis (Rcs) is one of the most complex signaling pathways in bacteria. It is structured around the RcsCDB His-Ass phosphorelay, but is controlled by upstream protein regulators, the sensory component RcsF and the phosphorelay inhibitor IgaA. The regulatory mechanism of Rcs is poorly understood. Over-activation of the stress response is lethal and, for this reason, igaA is essential unless any of the downstream components RcsCDB components are inactivated. While this genetic analysis cannot determine the direct target of the IgaA inhibition, the hypothesis was put forward that IgaA inhibits the RcsC kinase, as it initiates the phosphorelay. This hypothesis was never directly tested but propagated over the years so many times that the recent Rcs literature states it as a fact and not a hypothesis in the introduction and discussion sections. In this manuscript, Wall et al used protein interaction studies and extensive genetic analysis to demonstrate that the phosphotransfer protein RcsD, and not RcsC, is the direct target for IgaA inhibition. This study is significant because by experimentally challenging this old assumption, it revisits the regulatory mechanism of a major envelope stress response in enteric bacteria. It will be general interest to anyone who studies regulatory pathways.

My major comments aimed to strengthen and clarify some of the aspects of a proposed new signaling model and to improve flow and readability.

1. Lines 494-499: Authors suggest that the “strong periplasmic interaction provides an anchor for interaction with RcsD, but likely not the region in which signal is sensed”. In my opinion, the observation that overexpression of IgaA missing the periplasmic loop is not sufficient to make a statement about the sensing mechanism. It is common that a decrease in affinity can be overcome by an increase in concentration. I am surprised that there is no discussion about RcsF interaction with IgaA periplasmic loops in this context. In the simplest scenario, RcsF could compete with RcsD for IgaA binding and in that way constitute the “sensing” mechanism.
a) Does RcsF overexpression or inner membrane-mislocalized RcsF affect the IgaA/RcsD binding in BTH?
b) Is the strain overexpressing IgaA without the periplasmic loop competent for sensing? It can be constitutively OFF.

**Response**:

1. The discussion has been modified (now lines 447-462), to reflect the points raised by the reviewer and hopefully clarify our thinking.

a): While this is potentially an interesting experiment for future work, we have not added any experiments, primarily because we do not think that the BTH assays are the best way to look at this. The interactions in the bacterial two hybrid assays are from multicopy plasmids, and thus are generally not sensitive to modest changes in other cell constituents (for instance, inner membrane RcsF localization). We had earlier done experiments to ask if polymyxin treatment, for instance, was sufficient to show changes in the BTH interaction. We saw a two-fold increased (rather than the expected decreased) interaction between RcsD and IgaA, but this was seen as well in an *rcsF*- host, suggesting it did not reflect the usual pathway of polymyxin signal transduction. This data has not been included.

b) We have performed some additional experiments to address this suggestion and include them here for the reviewer. Because they do not provide new insight to an already complex paper, we did not include them in the paper (but can if requested). We used our T18-IgaA plasmids, since the parallel set of overproduction plasmids in the pBAD vectors were not available. A vector control, full-length IgaA or IgaA deleted for the periplasmic domain, under control of an IPTG-inducible promoter, were tested in a wild-type strain expressing the P_rprA_-mCherry reporter and a *lacI*^Q^ repressor; cells were grown with or without IPTG or PMBN and signal measured. The results are shown below. In the control (vector only), PMBN shows good induction of signaling, from the chromosomal Rcs components, as expected. Overproduction of IgaA (blue bars) increased the expression of the P_rprA_-mCherry by two-fold, for reasons we do not currently understand, but entirely blocked the response to PMBN. Overproduction of the construct deleted for the periplasm (green bars) had lost the inhibitory activity and had expression similar to the vector control. Note that we know that this IgaA-T18 derivative makes protein (Fig. S2H). In a model in which RcsF and IgaA interact via the IgaA periplasmic loop, extra IgaA in excess of RcsD might be expected to have this effect; we suggest that there is enough IgaA so that different molecules of IgaA are interacting with RcsF from those that are inhibiting RcsD. In that situation, the system will be relatively blind to sensing PMBN via RcsF. We are not surprised by any of this, because in our hands the periplasmic domain of IgaA is critical for the strong interaction with RcsD. We assume that chromosomal IgaA, even if not abundant, can provide wild-type interactions with RcsD and certainly with RcsF, and these then can respond to PMBN appropriately. While we did not think it was useful to add this data to the paper, the approach may prove valuable in further dissecting the interaction of IgaA with RcsF and RcsD, using point mutations or shorter deletions in the periplasmic domain, and we thank the reviewer for the suggestion.

2. Authors suggest that the RcsD “PAS” domain plays an important role in the IgaA-mediated regulation, mainly because of the expression of the RcsD N-terminal domain (periplasmic loops and transmembrane helixes) is not sufficient for a full display of phenotypes.
a) Is it possible that the cytoplasmic domains of RcsD play an indirect role by facilitating dimerization and thereby affecting the quaternary structure of the RcsD periplasmic domain? Many PAS domains also act as dimers.
b) The phenotype of the “PAS domain only” construct was not tested but is important for the overall conclusion of the paper. Is there a detectable interaction with IgaA in BTH or titration experiments? While there is no detectable interaction with “326” construct, as authors discussed, other cytoplasmic domains may be inhibitory.

Strain NM960 (MG1655 *lacI*^Q^, ∆*ara*714::P*_rprA_*-mCherry) was transformed with pUT18N, pEAW1 and pEAW1peri respectively. Colonies were grown overnight in MOPS minimal glucose media with 100µg/ml ampicillin. Cells were diluted to an OD_600_ ~ 0.05 in MOPS minimal 0.05% glucose 0.5% glycerol and ampicillin. Each strain (200µl) was loaded in triplicate into a 96 well plate. PMBN (40 µg/ml), IPTG (1mM) or both were added to different wells and the plate was incubated and read for 7h in a Tecan fluorescent plate reader. Bar graphs represent time point around OD 0.3 of average of triplicate with standard deviation.

**Response:** We suggested that the PAS-like domain was important for a variety of reasons. First, as the reviewer notes, is that the periplasmic loops/TM helices of RcsD are not sufficient for titration of IgaA in the presence of chromosomally encoded intact RcsD, and fail to interact with IgaA in the BTH assay, while the parallel constructs that extend to amino acid 461, containing in addition the PAS-like domain, both interact robustly and titrate (Fig. 2B, 3A, 3D). Therefore, minimally, all necessary regions for a strong enough interaction to drive titration are contained in the 1-461 region. We have now added additional tests of a newly made derivative of RcsD1-461 deleted for the periplasmic domain (RcsD_1-461,∆45-304_). This protein carries only the TM regions and the PAS-like domain. It interacts robustly with IgaA (Fig. 2C), strongly supporting a critical role for the PAS-like domain in mediating the interaction with IgaA. We believe this provides a better “PAS-only” construct than a soluble version of this domain; every soluble construct we tested was unable to interact with IgaA by the BACTH assay, suggesting an important role for membrane localization. As discussed further below, RcsD_1-461,∆45-304_ also supports a role for the periplasmic domain for titration, since it loses the ability to titrate IgaA (new Fig. 3C).

a. The reviewer asks if the PAS domain could act indirectly (only) by changing RcsD quaternary structure (leading to RcsD dimerization, possibly, I gather). We cannot fully rule this out, and now note that in the revised manuscript, although we believe the new data makes that a less likely role for this domain. Our bacterial two-hybrid tests of RcsD multimerization, not included in the manuscript, suggest the multimerization signal for RcsD/RcsD is not robust and that multiple regions are capable of promoting RcsD/RcsD interaction. Future work will be necessary to determine if RcsD does dimerize in its natural context and if that is important for signaling.

b) Testing PAS alone: As noted above, RcsD_1-461_, _∆45-304_ was created and compared to RcsD, RcsD_∆45-304_ and RcsD_1-461_ for interaction with IgaA. All of these interacted (Fig. 2C). We created this derivative because all soluble versions of RcsD we tested failed to interact with IgaA; these results further support a primary role for the PAS-like domain in driving the RcsD-IgaA interaction in the BACTH assays.

3. What is a specific model for how IgaA/RcsD interaction inhibits phosphorelay? Do authors think that RcsD is physically titrated away from RcsC or all proteins form a stable complex that undergoes the conformational change? Does RcsC/D interactions in BTH change in the absence of IgaA (in rcsB background)? It should be easy to test since authors have all of the constructs on hands. Regardless of the outcome, I think it will be valuable information for future mechanistic studies.

**Response**: We do not really know the answer to this. The experiment proposed by the reviewer will need to be done in some other fashion, since we are not able to detect the full-length RcsC interaction with RcsD in the BTH assay, even though it must interact under some conditions (to allow phosphate transfer from RcsC to the Hpt domain of RcsD). We have now added that negative data to S2D Fig. We also tested the RcsC deleted for the periplasmic domain, since that is functional (Fig. 6, Fig. S6B); it was also unable to interact with RcsD or IgaA. We cannot rule in or out that all three could interact simultaneously, but we have no direct data to address this. We note, however, that the interaction of RcsD and IgaA is unchanged by inactivating RcsC (S2B and S2C Fig), suggesting at least that all three do not need to be present in the cell to detect the interaction of RcsD and IgaA.

4. Authors performed very extensive analysis to test for IgaA/RcsD/RcsC interactions using bacterial-two hybrid (BTH), growth, signaling phenotype with many different alleles in various genetic backgrounds. Following these multiple phenotypes is quite challenging throughout the manuscript and requires constant flipping back and forth between text, figures, and extensive supplementary material. I suggest that adding a summary of all phenotypes for each of the mutant alleles in a table format would be helpful for readers. It will also enable authors to reference this table in the discussion section as opposed to referring back to the original multi-panel figures.

**Response:** We have now added a new figure panel, Fig. 3D, to summarize the critical data for the RcsD truncations.

5. Likewise, there are many genetic constructs with less conclusive phenotypes that are extensively discussed in the main text with references to the supplementary figures. These mutants will be a great asset for those working directly on Rcs; but, in my opinion, they often take away from the main message of the paper. While I let authors to decide which are the most critical to keep in the main text, I would encourage them to move as many details to the corresponding supplemental figure legends in order to preserve the flow of the manuscript.

**Response**: We have now shortened a number of sections of the text, moving the details to the supplemental figure legends and to a new supplemental text section. Sections that have been moved: 1) Lines 106-118 in original submission, about other *rcsD* alleles, moved to appropriate figure legends. 2) Lines 211-225 in original submission, about possibility of unexpected Hpt domain expression, now moved to S1 Text. 3) We removed panels S3E, F of the original submission, showing effects of overexpressing various RcsD derivatives on signaling and PMBN induction, as well as text describing this (lines 252-285). Some of this was redundant with other figures. Other parts, while still of significant interest, will require further work to clarify and interfered with the flow of the paper as reorganized.

Other specific comments:

Line 84: “Bar graphs of fluorescence at OD600 0.4.” is a sentence fragment.

**Response**: Corrected.

Lines: 139-144 and Fig. S2: What is the nature of a 4-fold cutoff?; it seems arbitrary. While small, there is a detectable interaction between IgaA and RcsC that seems to be RcsD-dependent and it looks significant based on the error bars.

**Response**: While we agree that the 4-fold cutoff is somewhat arbitrary, results were reproducible using this cutoff, interactions considered positive were generally seen in both orientations and signals we considered positive were, in most cases, well above the 4-fold cutoff. In Fig. S2D, the apparent best IgaA/RcsC interaction is 2x above the vector control, gives only 150 Miller units (very low in this assay), and is much less for the other orientation. The IgaA/RcsD interaction gives 1000 units, in either orientation, in parallel experiments. The low numbers are reproducible, but the nature of this assay is that it has a fair amount of variability and very low signals are dangerous to interpret as positive. Given our difficulty detecting any robust interaction by the full-length RcsC and by RcsC∆45-314, deleted for the RcsC periplasm (new S2D and S2E Fig data), we have modified our discussion in the text.

Line 169 (entire section). Are levels RcsD comparable? There is no associated immunoblot.

**Response**: The RcsD antibody we currently have does not do a good job of detecting the truncated RcsD constructs and we therefore cannot easily compare amounts in the pBAD-RcsD constructs used in Fig. 3. We have now replaced the Western blot in S2F Fig with the comparison shown below. The figure shows a comparison for the RcsD proteins expressed from a set of RcsD-T18 constructs, detected either with the anti-RcsD antibody (left panel) or the anti-CyaA antibody (right panel). From the Cya antibody results, most of the tested T18 fusion proteins are present at comparable levels, with the exception of pEAW7c, encoding RcsD_ABL-Hpt_. However, the RcsD antibody detection of the same proteins shows that all constructs that were missing the Hpt domain [7alpha (RcsD_1-522_), 7b (RcsD_1-683_), 7m (RcsD_1-383_] were poorly detected compared to those including that segment [7( RcsD), 7d (RcsD_Hpt_), 7s (RcsD_326-C_), 7peri (RcsD_∆45-304_)].

Comparison of RcsD polyclonal antibody to CyaA monoclonal antibody

NEB DH5alpha cells carrying a set of pEAW7 (RcsD-T18) plasmids were grown in LB Amplicillin at 37°C to an OD600 of 0.3, and induced with 1 mM IPTG for one hour. Equivalent samples were probed with the two antibodies. Plasmids: 7: RcsD+; 7alpha: RcsD_1-522_; 7b: RcsD_1-683_: 7c:RcsD_ABL-Hpt_ ; 7d: RcsD_Hpt_); 7m; RcsD_1-383_; 7s: RcsD_326-C_; 7peri: RcsD_∆45-304_.

From this data, we come to a few conclusions. First, the RcsD polyclonal antibody probably has important epitopes in the C-terminal domains of RcsD. Second, there are not drastic differences in expression levels at least in the T18 fusions, although WT is probably best expressed, and ABL-Hpt may lose the Cya tag. This is the best we can do for the moment in terms of quantifying the expression of the untagged versions used in Fig. 3. We would argue that it is not likely that the differences in behavior in Fig. 3 are due primarily to differences in levels, in part based on the RcsD-T18 quantitation, but also because the majority of these plasmids have phenotypes, suggesting protein is produced, in one strain or another. Only the 1-383 construct lacks a phenotype, and it appears to be expressed at a level equivalent to that for a number of the other constructs.

Lines 221-224 and Fig. S1E: Are there any smaller fragments in the RcsD immunoblot?

**Response**: This is one of the sections that was moved out of the main text and into supplemental material. In the new Western blot shown above, the T18 tag is at the C-terminus of RcsD, so that one might have seen such smaller fragments, but none are apparent in this experiment. Future experiments will include tagging the RcsD C-term to allow us to look directly, and further defining the region that may be necessary if such a fragment is made. However, given that this is not a major focus of this work, we have not expanded on this here.

Lines 388-392: Negative results here are inconclusive about the importance of the L643 for direct interactions. Ala substitution may not have a phenotype because of similar properties. I would just rephrase that L643P may either directly or indirectly affect binding.

**Response:** We do not fully agree, and are not sure we understand the concern. If we had just done alanine scans across this region (as we did, at least with single mutants) and found no disruption, we would most likely conclude that this was not a critical region. We looked at this part of the periplasmic loop because the L643P mutation, certainly a disruptive change, was one of the only alleles previously identified that made a stable protein, and we found that it did lose interaction in the bacterial two-hybrid assay. Therefore, we started these tests in the hope that this would define an interaction region. However, that is not what the additional alleles demonstrate. The site of the mutation, near the TM region, also makes it more likely to disrupt proper membrane insertion. This section has been somewhat reworded, and now reads (lines 352-357): “However, because other alleles at this position (L643A) or nearby mutations did not disrupt interaction or activity (S5D and S5E Fig), we conclude that L643 is not likely to directly participate in IgaA function or directly interact with RcsD. The introduction of proline in place of L643 may affect folding or localization of a critical region of IgaA, disrupting its ability to properly interact with RcsD. “

Lines 473: “A22 and Mecillinam, peptidoglycan disruptors”. Please rephrase as it implies physical disruption, which is not the case.

**Response:** This was rephrased (lines 427-429): “As previously described, inducers such as PMBN, believed to disrupt LPS interactions, A22, an inhibitor of MreB, and mecillinam, a beta-lactam antibiotic affecting peptidoglycan, act by changing the nature of the RcsF/IgaA interaction.”

Lines 530-534 and figure S6: I think the difference in conservation is very intriguing; is it possible to give some quantitative value to this observation? The figure needs to be properly explained. Also, I think it’s important to use rcsC and rcsD sequences from the same species representative to avoid the sequence bias. Why was the different number of RcsC and RcsD sequences used?

**Response:** Fig. S6 (now Fig. S7) has been replaced with a version with the same species shown for the two proteins and the legend expanded. However, we have not added quantitative values.

Figure 1A: This figure is reused from the recent review article by the same authors. Please verify the permissions for that.

**Response:** While this is similar to the figure in the review, it is not the same one. Both are derived from our (previously unpublished) starting figure. The figure has been changed a bit more (we removed *flhDC*, not relevant here).

Figure 4C: I don’t think it’s essential and can be moved to the supplement.

**Response:** We don’t agree on this point. We don’t present quantitative data on what we consider very important observations because of the very rapid accumulation of suppressing mutations in these strains. Given that, the plate assay provides what we think is rather striking data showing the differences in roles for different regions of IgaA. We have reworded text in this section (lines 363-380) and hope this clarifies why we believe this is critical data.

Reviewer #2: The Rcs phosphorelay is a particularly complex stress signaling system that senses and responds to envelope damage. Stress conditions change the interaction between the outer membrane stress sensor lipoprotein RcsF and the essential inner membrane protein IgaA, which in turn impacts the interaction between IgaA and the downstream components of the phosphorelay, turning Rcs on. How IgaA interacts with the phosphorelay and controls its activity was unknown. In this article, Wall and coauthors use an elegant combination of in vivo interaction assays (bacterial adenylate cyclase two hybrid assay – BACTH), genetic analysis, mutagenesis, phenotypic experiments and reporter assay experiments to investigate the interaction between IgaA and the two other inner membrane components of Rcs, the histidine kinase RcsC and the phosphotransfer protein RcsD. They found that, in contrast to what was generally assumed, IgaA regulates Rcs by interacting with RcsD and not with RcsC, although an IgaA-RcsC interaction cannot be ruled out. Using RcsD and IgaA fragments, they also dissected the interaction between the different domains of these two proteins. In sum, their results reveal that the periplasmic domain of IgaA functions as an anchor for the periplasmic domain of RcsD and that the periplasmic contact between these two proteins is necessary for repression. Crucial, although weaker, interactions also take place in the cytoplasm; these contacts, in particular with loop 1 of IgaA, are necessary for the regulation of Rcs. Additional experiments aiming at understanding the RcsD-RcsC interaction are also reported.

Overall, the paper is interesting and provides an important and novel contribution to the understanding of the particularly complex Rcs system. An impressive amount of data is presented: these data convincingly support the proposed model that the IgaA-RcsD interaction is crucial in controlling Rcs activity, which is a major step forward. The data are adequately discussed and thoroughly interpreted.

Here are my comments:

1-the paper, although well and nicely written, is extremely long, in particular the results section; this section describes key data, which are presented in the main manuscript, and that are central to the message of the paper. However, less important experiments, whose results are presented in the supplementary material, are also described at length. As a result, the reader loses focus and gets distracted, which makes the reading of the manuscript more challenging. I would suggest moving some of the description of the less crucial experiments to the supplementary material, to help the reader understanding the key message of the paper.

**Response**: As noted in the response to reviewer 1, a number of sections have been moved as suggested: 1) Lines 106-118 in original submission, about other *rcsD* alleles, moved to appropriate fig. legends. 2) Lines 211-225 in original submission, about possibility of unexpected Hpt domain expression, now moved to S1 Text. 3) S3E and S3F Figs and Lines 264-285 in original submission, about unexpected signaling from some RcsD constructs, were removed from the paper). Panel S2G in the original paper was also removed. In addition, there has been significant rewriting to hopefully clarify and we have added a new panel (Fig. 3D), summarizing the results of our dissection of RcsD, to help the reader.

2. the results of the BACTH assays are convincing when an interaction is detected. However, the absence of signal does not provide an evidence that the tested partners do not interact. This is particularly true when it comes to the RcsC-IgaA potential interaction. To that respect, an important control is missing: the authors should test whether they can “detect” the RcsC-RcsC dimer , which would indicate that the RcsC constructs are working in the BACTH assay.

**Response:** We very much agree that a negative interaction is not full evidence of lack of interaction. We have looked further to see if we can detect the type of positive control requested. As now shown in new panels in S2D Fig, we were unable to detect RcsC/RcsC or RcsC/RcsD interactions. Because the RcsC plasmids tended to interfere with cell growth, we also tested an RcsC derivative deleted for the periplasmic region (RcsC_∆45-314_), hoping this would be less toxic (S2E Fig). The chromosomal version of this allele is fully functional in the phosphorelay (Fig. 5) and responds to PMBN induction. Unfortunately, we are unable to detect any significant interactions of this with RcsD, or with IgaA (S2E Fig). However, please note that the parallel RcsD construct (RcsD_∆45-304_) does interact well with IgaA (Fig. 2B). We had previously found that RcsC_326-C_, encoding the soluble portion of RcsC, does interact robustly with RcsD (Fig. S6A). We have now replaced this with a new experiment, showing the same interaction but also demonstrating that this too is negative for interaction with IgaA (new S6A Fig). However, since the equivalent RcsD construct, RcsD_326-C_, also does not interact with IgaA, this result may not tell us much. Based on all of these attempts and negative results, we have rephrased the text throughout the paper to note the caveats, emphasizing that while we have no evidence of direction interaction of IgaA and RcsC, we cannot fully rule it out.

Reviewer #3: In this manuscript, the authors investigate the interactions between the Rcs signaling proteins which control an envelope stress response in Gram negative enteric bacteria. The provide evidence that the “brake” protein IgaA interacts with the membrane bound histidine phosphotransfer protein RcsD, but not the histidine kinase signaling initiator RcsC. They localize this region, using the BACTH assay to a putative PAS motif in the cytoplasm, but not containing the histidine phosphotransfer domain. They go on to show that RcsD constructs interacting with IgaA also have the ability to activate the wild-type Rcs pathway when expressed in trans, suggesting they have the ability to titrate the negative regulator IgaA away from the wild-type signaling complex. The authors go on to isolate a mutant in the RcsD PAS domain that both dampens signaling in response to inducing cues and appears to strengthen IgaA interaction through cytoplasmic domain 1 of IgaA. The RcsD PAS domain mutant is capable of diminishing Rcs signaling in strains containing IgaA cytoplasmic loop 1, further supporting this interaction. Finally, the authors go on to demonstrate that the periplasmic loop of RcsC is not involved in signaling in response to a number of envelope stresses, and suggest that RcsD interaction with IgaA is instead the point of signal reception in these cases.
This is a dense paper with a wealth of genetic data that add to our understanding of how the Rcs signaling cascade works. None-the-less, it was somewhat difficult to follow the thread of the story due to the sheer amount of data presented and the fact that it was split between the manuscript itself and the supplemental data. The genetic data supports the conclusions regarding RcsD:IgaA interaction, but it would be nice to have data demonstrating the physical interactions to validate the genetics. There are some inconsistencies in the data that the authors do a good job trying to address, but one also wonders whether these inconsistencies reflect unknown aspects of interactions between RcsD and RcsC or other proteins. This possibility is not really addressed.

**Response:** We thank the reviewer for the thought-provoking questions raised here and below. We hope that moving some material into the supplemental will make the main paper easier to digest. Certainly there are many aspects of this system we do not yet understand, and we are continuing to pursue this. Possibly because of the multipass membrane characteristics of IgaA and RcsD, our attempts thus far and with expert collaborators to get clear evidence of a physical interaction have not been successful. We continue to pursue this, but it is well beyond the scope of this paper, given the current state of working with these proteins. We certainly cannot rule out other players, but do not see any basis to invoke these at this point.

Other Comments:
1. Line 84 – there is some sort of typo in this line. The sentence “Bar graphs of fluorescence at OD600 0.4” doesn’t fit.

**Response**; Rewritten: “Fig 1B shows a bar graph of the fluorescence for cells at OD_600_ 0.4 when wild-type and mutant cells are exposed to a small molecule stimulator of Rcs signaling, the non-toxic cationic polymyxin B nonapeptide (PMBN).”

2. Fig. 2B – it is a bit perplexing that the authors argue for an interaction between the periplasmic domain of RcsD and IgaA based on this data, since the construct lacking the cytoplasmic domain is one of the weakest interactors in this assay.

**Response**: Revisiting this, we agree with the reviewer that the bacterial two hybrid data in Fig. 2B does not provide good evidence for the interaction of the periplasmic domains; this question prompted us to explore this further. New data has now been added (Fig. 2C), showing evidence for interaction of RcsD_1-461, ∆45-304_ with IgaA, further suggesting that the PAS-like domain rather than the periplasmic domain drives the interaction here. We have rephrased this part of the paper, and now only argue for an important periplasmic contact based on the behavior of various constructs in Fig. 3 and later in the paper.

3. Fig. 3A – why does over-expression of WT RcsD not have a stimulatory effect? This protein binds to IgaA as demonstrated in the BACTH, and one would expect that over-expression should titrate out IgaA and lead to a similar stimulatory effect as the other constructs that interact with IgaA?

**Response**: We don’t fully know, and continue to investigate this. Most likely is that IgaA interaction with full-length RcsD results in an RcsD that is acting to move phosphate from RcsB back to RcsC. Only when that cannot happen (deletion of the regions involved in this phosphatase cascade) do we see titration.

4. It is unclear what the experiments in Fig. 3B add. The negative data in the wild-type are consistent with previous conclusions that a loss of interaction with IgaA is correlated with a loss of the ability to interfere with wild-type signaling, but the data in the right panel only serve to underscore that in the presence of a mutant (very lowly expressed?) version of RcsD, the Hpt domain in these constructs is capable of moving phosphate through the signaling pathway. This data is a bit distracting from the main points the authors try to make regarding IgaA:RcsD interaction.

**Response:** In the somewhat reorganized version of the paper, the data in this figure becomes particularly important in supporting the importance of the RcsD periplasmic domain in allowing repression by IgaA. RcsD_∆45-304_ differs from RcsD only by loss of the periplasmic domain, but now signals constitutively, suggesting loss of a critical interaction with IgaA. This is also supported by the poor growth of cells carrying this plasmid, even without induction, and our inability to introduce this mutant into the chromosome. This whole section has been rewritten and we hope this new version is clear. We also added Figure 3D summarizing the phenotypes of various mutants, to put these mutants into context.

5. Line 176 “… expressed AT levels….”

**Response**: Corrected.

6. Lines 249-251: The authors state that “Finally, because this signaling is not seen in an rcsD+ strain, signaling by these fragments of RcsD is recessive to the full-length protein.” Do they interpret this to mean that the RcsD derivatives do not interact with RcsC as well as WT?

**Response:** That is certainly likely. Consistent with that idea is that a strain expressing the ABL-Hpt domains from the chromosome does not lead to the lethally high level of signaling that is seen with wild-type in the absence of IgaA. As noted in the response to reviewer 1 and 2, our tools for measuring RcsC interactions are not yet satisfactory, so this will need to wait for future work.

7. Lines 313-315: The authors state that “Thus, while rcsD326-C was negative in the bacterial two hybrid interaction with IgaA (Fig 2B), the continued dependence on IgaA for viability is consistent with it retaining a critical contact with IgaA”. Could some of the inconsistencies between the interaction with IgaA observed in the BACTH and the genetic/complementation results also be due to altered interactions between RcsD and RcsC, which are not investigated here?

**Response**: That may certainly be the case; at some level RcsD must change its interaction with RcsC depending on signal/IgaA status, and this will be a focus of future work. However, the apparent inconsistency of the behavior of the soluble portion of RcsD (no interaction but dependence on IgaA) is, we think addressed by the added new data, showing that a construct containing trans-membrane regions and the PAS-like cytoplasmic domain (Fig. 2C) still retains a strong IgaA interaction. This suggests that the IgaA/RcsD BACTH signal requires that both be at the membrane, and does not require that we invoke a lost interaction of RcsC and RcsD, for instance, for the requirement for IgaA. We hope the rewritten text is clear on this point.

8. Ruling out an RcsC:IgaA interaction based solely on an artificial BACTH assay seems premature?

Response: We definitely agree, particularly because the BACTH-RcsC constructs made cells quite sick. We have looked further at this, and added new results (all negative, however) to the paper in Fig. S2. In the revised paper, we have rephrased text to try to clarify that we cannot fully rule out an interaction, although we have no evidence that one exists. Certainly the interaction that might have seemed most likely, between the periplasmic domains of RcsC and IgaA, can be ruled out by our finding that deletion of the RcsC periplasmic domain does not interfere with the response to PMBN (Fig. 5B) or make cells able to accept a deletion of igaA (Fig. S4D).

9. There is a lot of text at the beginning of the paper and in the supplementary data devoted to discussing the development of an rprA-mCherry reporter, which appears to behave very similarly to other previously published rprA reporter genes. Since the manuscript is quite long, the authors might consider condensing the discussion of the data presented around this topic.

**Response**: We have moved some of this material into the supplemental legends, but did want to confirm that this assay reflects everything we know about signaling in this phosphorelay.

10. A nice further validation of the authors’ model for IgaA:RcsD contacts would be to look at the impact of both the T411A and periplasmic loop mutations together. The model presented here would suggest that these mutations would be additive.

**Response:** We have not yet made this construct. Our expectation from the experiment in Fig. 4C is that in the absence of the periplasmic loop of IgaA, T411A is unable to effectively suppress the loss of periplasmic part of the IgaA/RcsD interaction. We plan to look at point mutations in the RcsD periplasmic loop (rather than full loss of the region), and agree it would be good to examine how T411A affects their phenotype.

**Have all data underlying the figures and results presented in the manuscript been provided?**
Large-scale datasets should be made available via a public repository as described in the *PLOS Genetics* [data availability policy](http://journals.plos.org/plosgenetics/s/data-availability), and numerical data that underlies graphs or summary statistics should be provided in spreadsheet form as supporting information.

Reviewer #1: Yes

Reviewer #2: Yes

Reviewer #3: Yes
